# Usefulness of Artificial Intelligence for Transnasal Esophagogastroduodenoscopy in Clinical Practice

**DOI:** 10.7759/cureus.111684

**Published:** 2026-06-28

**Authors:** Eisuke Nakao, Satoshi Asai, Ayumu Chaen, Tomoya Hashimura, Kento Hisamatsu, Yuma Fujita, Shuta Otachi, Kenji Matsuo, Kotaro Takeshita, Eisuke Akamine

**Affiliations:** 1 Gastroenterology, Tane General Hospital, Osaka, JPN; 2 Gastroenterology, Nakagami Hospital, Okinawa, JPN; 3 Gastroenterology, Rokko Hospital, Kobe, JPN

**Keywords:** artificial intelligence, clinical practice, gastric cancer, negative predictive value, transnasal esophagogastroduodenoscopy

## Abstract

Objectives

We evaluated the diagnostic performance of an artificial intelligence (AI) diagnostic system for biopsy-considered suspicious lesions during transnasal esophagogastroduodenoscopy (EGD), focusing on its negative predictive value (NPV) to demonstrate whether a negative AI result may help endoscopists make a more conservative biopsy decision in selected low-risk lesions.

Methods, patients, and materials

We conducted a prospective single-center observational study. Endoscopists observed the entire stomach using transnasal EGD and the AI diagnostic system (gastroAI™ model-G, AI Medical Service Inc, Tokyo, Japan). Two endoscopic images of the suspected lesion were recorded using an AI diagnostic system; biopsies were collected for histological diagnosis, regardless of the AI diagnosis. The primary endpoint was the NPV of the gastroAI™ model-G. The reference standard used for calculating the diagnostic performance of the gastroAI™ model-G was histopathological diagnosis.

Results

Overall, 392 patients were enrolled, and 98 specimens from 87 patients were analyzed. A total of 11 endoscopists (five experts and six non-experts) performed the examinations. Five specimens (5%) were diagnosed as adenocarcinoma. As all specimens diagnosed as “negative” using the gastroAI™ model-G were pathologically benign, the NPV of the system was 100% (95% confidence interval (CI): 84%-100%). Its sensitivity, specificity, positive predictive value, and accuracy were 100% (95% CI: 36%-100%), 34% (95% CI: 25%-45%), 8% (95% CI: 3%-17%), and 38% (95% CI: 28%-48%), respectively. Specificity and accuracy were significantly higher for endoscopists than for the model (96% vs. 34% and 94% vs. 38%, respectively; p < 0.01), whereas the model had a higher sensitivity than did endoscopists (100% vs. 60%), although the difference was not statistically significant.

Conclusions

This study reveals the potential clinical utility of the AI diagnostic system for transnasal EGD, particularly indicating its high NPV.

## Introduction

Gastric cancer has a high survival rate when detected at an early stage; however, the survival rate becomes lower than that of other cancers as the stage advances [1]. Therefore, early detection and treatment are crucial for improving prognosis. Screening esophagogastroduodenoscopy (EGD) plays an important role in its early detection. Based on a large case-control study, patients who underwent gastric cancer screening at least once every five years had a 47% reduction in associated mortality compared with those who had never been screened [2].

Transnasal EGD is often used for screening during medical checkups owing to concerns regarding patient tolerance [3]. Although transnasal EGD is less invasive than transoral EGD, it lacks magnification capabilities and offers lower image quality. Therefore, the diagnosis of any suspected cancerous area relies heavily on biopsy findings. However, unnecessary biopsies should be avoided not only for cost-effectiveness but also because of the risk of delayed bleeding in screening EGD.

Recently, artificial intelligence (AI)-based diagnostic support systems using deep learning in gastrointestinal endoscopy have shown remarkable progress. These advances began with their effectiveness in the detection of colorectal polyps [4] and neoplastic lesions using upper gastrointestinal endoscopy [5-9]. However, almost all previous studies on AI diagnostic support systems for EGD have been conducted at specialized facilities using transoral EGD; moreover, the effectiveness of these systems in transnasal endoscopy has not been previously demonstrated. Additionally, although the sensitivity is sufficiently high, a high false-positive rate remains a problem [10]. Furthermore, all existing studies have focused on lesion detection, specifically sensitivity; the ability to correctly diagnose non-lesions as non-neoplastic has not been addressed, indicating a negative predictive value (NPV). If the NPV is sufficiently high, unnecessary biopsies can be avoided, leading to effective screening during EGD.

In 2024, the gastroAI™ model-G (AI Medical Service Inc, Tokyo, Japan), an AI diagnostic support system that indicates whether additional biopsy is necessary, was launched. The diagnostic accuracy of this AI diagnostic system has been demonstrated to be comparable to that of specialists [11], and the system is currently being applied in clinical practice. Its application to transnasal endoscopy, a procedure frequently used for screening, can help endoscopists make a more conservative biopsy decision in selected low-risk lesions and potentially enable safer and more cost-effective endoscopic examinations. In this study, we aimed to evaluate the diagnostic performance of the gastroAI™ model-G for transnasal EGD.

## Materials and methods

Study design and ethical considerations

This prospective single-center observational study was conducted at Tane General Hospital from May 2024 to October 2024 and approved by the Institutional Review Board of Tane General Hospital (IRB number: 2024-04). This study was registered with the University Hospital Medical Information Network (UMIN) Center (UMIN: 000054282). Written informed consent was obtained from all participants after explanation of the study protocol.

Patients

Patients undergoing transnasal EGD for screening or surveillance at our hospital between May and October 2024 were enrolled in this study. We included those aged ≥20 years and who provided informed consent. The exclusion criteria were as follows: (1) history of total gastrectomy; (2) contraindication for biopsy; (3) pregnancy; (4) comorbidity of psychosis, psychiatric symptoms, or dementia; and (5) ineligibility for registration, as judged by the attending physician. All EGD procedures and AI monitoring information were recorded on video, and all endoscopic images were documented.

Endoscopic procedures

The endoscopists observed the whole stomach using white light imaging. Biopsy specimens were obtained based on the following criteria: all lesions (excluding known lesions) identified by the endoscopists, including benign and malignant lesions, which the endoscopists determined required biopsies. Two endoscopic images of the lesions were recorded using the AI diagnostic system. Endoscopists collected biopsies for histological diagnosis irrespective of the AI diagnosis results. In this study, lesions were classified as “malignant” if the endoscopists suspected malignancy with high confidence and “benign” for other cases. All examinations were performed using the EVIS X1 processor, a light source (Olympus Medical Systems, Tokyo, Japan), and transnasal scopes, such as GIF-XP260NS, GIF-XP290N, or GIF-1200N (Olympus Medical Systems).

Outcome measures

Endoscopists assessed suspicious lesions and collected information, including the pre-biopsy diagnosis (malignant or benign), location, size, morphology, observation time, and adverse events. Pre-biopsy diagnoses were classified based on the confidence level of the endoscopist and defined as “endoscopists’ diagnosis.” Lesions for which the endoscopist strongly suspected malignancy prior to biopsy were classified as “malignant,” whereas those for which the endoscopist determined that a biopsy was necessary to rule out malignancy were classified as “benign.” Lesions observed before examination were excluded from the analysis. After examination, the biopsy specimens were histopathologically diagnosed and evaluated by a pathologist. The endoscopists participating in this study were divided into two groups: (1) experts, defined as those certified by the Japanese Gastroenterological Endoscopy Society, and (2) non-experts, comprising the rest of the endoscopists.

AI diagnostic system

**Figure 1:**
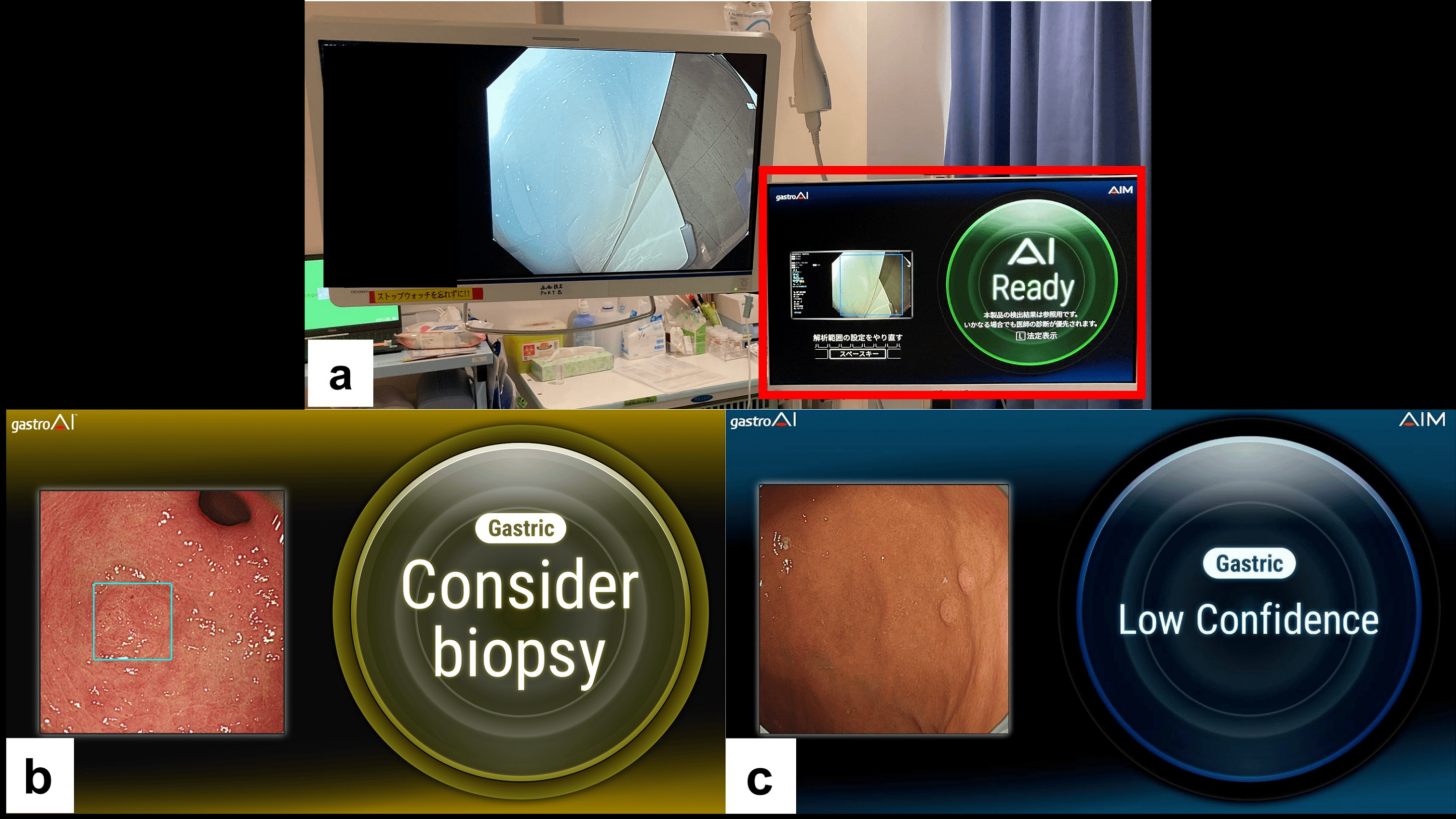
The gastroAI™ model-G (artificial intelligence (AI) diagnostic system) (a) The monitor display, where the red rectangle indicates the AI monitor; (b) a rectangular display notifies “consider biopsy” to the endoscopists when a suspicious lesion requiring additional examinations is detected; (c) when no suspicious lesions are detected, the AI analysis result is displayed as “low confidence.”

The gastroAI™ model-G is a real-time automatic detection system for gastric epithelial lesions developed by AI Medical Service Inc (Figure 1). When endoscopists freeze an endoscopic image, the AI analysis results are displayed on the monitor. A rectangular display notifies the endoscopists with the message “consider biopsy” when a suspicious lesion requiring additional examinations is detected. When no suspicious lesions are detected, the AI analysis result is displayed as “low confidence.” We defined a lesion as “positive” if the AI suggested “consider biopsy” in at least one of the two instances and “negative” if it indicated “low confidence” in both.

Endpoints

The primary endpoint was the NPV of the gastroAI™ model-G for transnasal EGD, defined as the ratio of the number of lesions diagnosed as negative by the AI to the number of lesions histopathologically diagnosed as benign. The secondary endpoints included sensitivity (the ratio of the number of lesions histopathologically diagnosed as malignant to the number of lesions diagnosed as positive by the AI), specificity (the ratio of the number of lesions histopathologically diagnosed as benign to the number of lesions diagnosed as negative by the AI), accuracy (the ratio of the total biopsy number to the number of correct diagnosis by the AI), positive predictive value (PPV; the ratio of the number of lesions diagnosed as positive by the AI to the number histopathologically diagnosed as malignant), total observation time, and adverse events. Total observation time was defined as the duration from scope insertion to its removal. Adverse events were defined according to the Common Terminology Criteria of Adverse Events version 5.0 [12].

Sample size calculation

As this was a single-center observational study and there are no previous studies on the efficacy of the AI diagnostic system in transnasal endoscopy, the sample size was determined based on the number of cases that were obtained at our hospital during the study period. The number of examinations performed per day in one room of our hospital was approximately 3-5, which was estimated to be 15-20 per week, 70 per month, and 420 every six months. Considering ineligible and dropout cases, the expected number of cases was set at 400.

Statistical analysis

All statistical analyses were performed using EZR version 1.70 [13], a modified version of the R Commander software designed to add statistical functions that are frequently used in biostatistics. Continuous and categorical variables were compared using the Mann-Whitney U test or chi-squared test. Statistical comparisons between the diagnosis by the AI system and that by the endoscopists were conducted using McNemar’s test for sensitivity, specificity, and accuracy. The significance threshold was set at p < 0.05.

## Results

Patient flow diagram and patients’ characteristics

**Figure 2:**
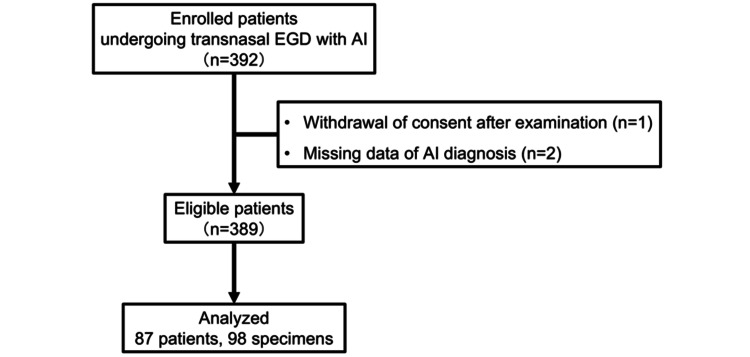
Patient flow diagram Multiple biopsy specimens were obtained from the same patient. EGD: esophagogastroduodenoscopy; AI: artificial intelligence

Overall, 392 patients were enrolled. Three patients were excluded: one withdrew consent after examination, and two had missing data on AI diagnosis (n = 2). Among 389 eligible patients, 98 specimens from 87 patients were analyzed (Figure 2).

Table 1 shows the characteristics of the study participants. The median age was 55 years (range, 29-88 years), with 259 (67%) male individuals and 122 *Helicobacter pylori* infection cases (31%). Fourteen (4%) patients were administered antithrombotic agents. Almost all endoscopic procedures were performed using GIF-XP290N or GIF-1200N, except for two cases where GIF-XP260NS was used. Five expert endoscopists examined 114 (29%) cases; the remaining 275 (71%) cases were examined by six non-expert endoscopists.

**Table 1:** Patients’ characteristics (n = 389) *A total of 11 endoscopists performed the examinations; five were experts, and six were non-experts.

Variable	Value
Number of patients, n	
Male	259
Female	130
Age, median (range) (year)	55 (29-88)
*Helicobacter pylori* infection, n (%)	122 (31)
Antithrombotic agents, n (%)	14 (4)
Scope, n (%)	
GIF-XP260NS	2 (1)
GIF-XP290N	209 (54)
GIF-1200N	178 (45)
Number of examinations, n (%)*	
Expert	114 (29)
Non-expert	275 (71)

Details of the examination

Table 2 presents the details of this study. The median observation time was seven (range: 4-18) minutes. Biopsies were performed on 87 (22%) patients, and 98 specimens were collected. The AI system diagnosed 66 (67%) and 32 (33%) biopsy cases as positive and negative, respectively, whereas endoscopists diagnosed seven (7%) and 91 (93%) cases as malignant and benign, respectively. Among the 98 (5%) specimens, five were diagnosed as adenocarcinoma, and the remaining specimens were diagnosed as follows: gastritis, 83 (85%); gastric ulcer, five (5%); and others, five (5%). No adverse events were observed. Tables 3, 4 show the contingency tables of the results.

**Table 2:** Details of the examination *Lesions for which the endoscopist strongly suspected malignancy prior to biopsy were classified as “malignant,” whereas those for which the endoscopist determined that a biopsy was necessary to rule out malignancy were classified as “benign.”

Variable	Value
Observation time, median (range) (min)	7 (4-18)
Case of biopsy, n (%)	87 (22)
Number of biopsies, n	98
Endoscopists’ diagnosis*, n (%)	
Malignant	7 (7)
Benign	91 (93)
AI diagnosis, n (%)	
Positive	66 (67)
Negative	32 (33)
Histological findings, n (%)	
Adenocarcinoma	5 (5)
Gastritis	83 (85)
Gastric ulcer	5 (5)
Others	5 (5)
Adverse events, n (%)	0 (0)

**Table 3:** 2 × 2 contingency table of the artificial intelligence system and histopathological diagnosis

	Histopathological diagnosis			
AI diagnosis		Malignant	Benign	Total
	Positive	5	61	66
	Negative	0	32	32
	Total	5	93	98

**Table 4:** 2 × 2 contingency table of the endoscopists’ diagnosis and histopathological diagnosis

	Histopathological diagnosis			
Endoscopists’ diagnosis		Malignant	Benign	Total
	Positive	3	4	7
	Negative	2	89	91
	Total	5	93	98

Diagnostic ability of the AI system

Table 5 presents the diagnostic ability of the AI system. The NPV of the AI system, the primary endpoint of this study, was 100% (95% confidence interval (CI): 84%-100%). Its sensitivity, specificity, accuracy, and PPV-the secondary endpoints of this study-were 100% (95% CI: 36%-100%), 34% (95% CI: 25%-45%), 38% (95% CI: 28%-48%), and 8% (95% CI: 3%-17%), respectively.

**Table 5:** Diagnostic ability of the artificial intelligence system based on histopathological findings CI: confidence interval; NPV: negative predictive value; PPV: positive predictive value

Metric	Value (95% CI)
NPV	100% (84%-100%)
Sensitivity	100% (36%-100%)
Specificity	34% (25%-45%)
Accuracy	38% (28%-48%)
PPV	8% (3%-17%)

The sensitivity, specificity, and accuracy of the endoscopists’ diagnoses were 60% (95% CI: 15%-95%), 96% (95% CI: 89%-99%), and 94% (95% CI: 87%-98%), respectively. Specificity and accuracy were significantly higher for endoscopists than for the AI diagnostic system (p < 0.01); nonetheless, the AI system had a higher sensitivity, although the difference was not significant (Table 6).

**Table 6:** Comparison of diagnostic ability between the AI and the endoscopist AI: artificial intelligence; NA: not applicable *McNemar’s test

	AI	Endoscopist	p-value*
Sensitivity	100% (36%-100%)	60% (15%-95%)	NA
Specificity	34% (25%-45%)	96% (89%-99%)	<0.01
Accuracy	38% (28%-48%)	94% (87%-98%)	<0.01

## Discussion

In this study, we revealed the diagnostic accuracy of an AI diagnostic support system for biopsy-considered suspicious lesions during transnasal EGD in clinical practice. This is the first prospective report investigating the clinical utility of AI in this context. The NPV of the AI system was the primary study endpoint, with a value of 100% indicating that a negative AI result may help endoscopists make a more conservative biopsy decision in selected low-risk lesions in clinical practice. These results are important for the future use of AI diagnostic support systems.

Hirasawa et al. first reported the efficacy of an AI diagnostic support system for detecting gastric cancers [14]. Subsequently, several investigations of gastric neoplasm detection using AI-assisted endoscopic systems demonstrated their superiority to endoscopists' detection [5-9,15,16]. However, most of these studies were retrospective in design, involved the use of endoscopic still images or endoscopically recorded movies, and were conducted at specialized institutions. Some randomized controlled trials [6-8] have shown a good diagnostic performance of these AI support systems for detecting gastric neoplasms. Although these studies provided sufficient evidence to apply the AI diagnostic support system in clinical practice, their usefulness for transnasal EGD remains unclear because transoral EGD was used in all previous studies.

Transnasal EGD is widely used in physical examinations and screening because it is highly tolerated by patients [17]. However, it offers inferior image quality compared with transoral EGD, and it is not suitable for detailed examination or surveillance of patients at high risk. Moreover, endoscopists rely heavily on biopsies because of the lack of magnification and light intensity reduction under image-enhanced endoscopy. However, unnecessary biopsies should be avoided-particularly during physical examinations or screening-to minimize post-biopsy bleeding and consider cost-effectiveness. It is possible to detect lesions and reduce unnecessary biopsies with the support of the AI diagnostic support system.

In this study, the NPV of the AI diagnostic support system was used as the primary endpoint to determine whether a negative AI result may help endoscopists make a more conservative biopsy decision in selected low-risk lesions. An NPV of 100% indicated that endoscopists could make a more conservative biopsy decision for suspicious lesions when the AI system suggested “low confidence” based on high-quality endoscopic images. Furthermore, the 100% sensitivity indicated adequate lesion detection. However, its specificity and PPV were only 34% and 8%, respectively, which were considered insufficient. When the AI diagnosis showed “consider biopsy,” the endoscopist’s diagnosis must be considered to determine if conducting a biopsy is appropriate. In this study, we primarily compared the diagnostic accuracy of endoscopists and the diagnostic performance of the AI support system as a sub-analysis. Similar to a previous study [15], our study showed that endoscopists demonstrated superior specificity and PPV, whereas the AI support system showed superior sensitivity, although this difference was not significant. Even when the analysis was limited to non-expert endoscopists, the results were similar, indicating that endoscopists and AI detection can complement the shortcomings associated with either, enabling higher-quality endoscopic examinations.

The AI diagnostic support system used in this study (gastroAI™ model-G) was not a real-time diagnostic device, as it displayed results 2 s after freezing. Additionally, because the endoscopists only referred to the AI diagnosis for areas suspected of being neoplastic, this did not lead to an extension in the examination time. Consulting an AI diagnosis for every endoscopic image is considered impractical, as it can potentially prolong the examination time and disrupt the endoscopist’s concentration. Therefore, it is presently considered appropriate to refer to an AI diagnosis only when neoplastic lesions are suspected, as in this study. Nevertheless, several studies indicate the effectiveness of AI-based real-time diagnosis in clinical practice, although the AI systems used differ from the one in the present study [5-9]. However, all previous studies were conducted in specialized institutions using transoral endoscopy; there are no reports from small-to-medium-sized hospitals or private clinics, nor any studies evaluating its efficacy in transnasal endoscopy. As the diagnosis of early gastric cancer is highly dependent on the experience of the endoscopist [18], AI support is expected to be particularly effective for less experienced endoscopists. Thus, studies using AI for real-time diagnosis in clinical settings are required.

This study had some limitations. First, this was a single-center, single-arm study with a relatively small sample size. Second, the final decision to conduct a biopsy was at the endoscopist’s discretion, resulting in the inability to examine missed lesions or accurately identify false positives of the AI diagnostic support system. However, in clinical practice, the endoscopist makes the final decision regarding biopsy, as in the present study. Therefore, the design was considered reasonable and reflective of real-world clinical practice. Third, the endoscopist’s diagnosis was categorized as “benign” or “malignant” based on the confidence level. However, as the possibility of malignancy was considered, to some extent, at the time of biopsy, it may not accurately reflect the diagnostic ability of the endoscopists.

## Conclusions

This study revealed the potential clinical utility of an AI diagnostic support system for transnasal EGD. The high NPV of the AI system indicated that a negative AI result may help endoscopists make a more conservative biopsy decision in selected low-risk lesions in clinical practice. Although further multicenter studies with larger sample sizes are needed, we believe that these findings will provide opportunities for the adoption of AI diagnostic support systems in small-to-medium-sized hospitals and private clinics.
